# A Miniature Eight-Port Antenna Array Based on Split-Ring Resonators for 5G Sub-6 GHz Handset Applications

**DOI:** 10.3390/s23249734

**Published:** 2023-12-10

**Authors:** Jianlin Huang, Lingrong Shen, Shanshan Xiao, Xiaojing Shi, Gui Liu

**Affiliations:** College of Electrical and Electronic Engineering, Wenzhou University, Wenzhou 325035, China; 194511981414@stu.wzu.edu.cn (J.H.); 21451841021@stu.wzu.edu.cn (L.S.); shi@wzu.edu.cn (X.S.)

**Keywords:** 5G, MIMO antenna, C-band, sub-6G antenna, split-ring resonator, Wang-shaped radiator

## Abstract

In this article, a miniature eight-port multiple-input multiple-output (MIMO) antenna array is proposed for fifth-generation (5G) sub-6 GHz handset applications. The individual antenna element comprises a radiator shaped like the Chinese character “王” (phonetically represented as “Wang”) and three split-ring resonators (SRR) on the metal frame. The size of the individual antenna element is only 6.8 × 7 × 1 mm^3^ (47.6 mm^3^). The proposed antenna element has a −10 dB impedance bandwidth of 1.7 GHz (from 3.3 GHz to 5 GHz) that can cover 5G New Radio (NR) sub-6 GHz bands N77 (3.3–4.2 GHz), N78 (3.3–3.8 GHz), and N79 (4.4–5 GHz). The evolution design, the current distribution, the effects of single-handed holding, and the analysis of the parameters are deduced to study the approach used to design the featured antenna. The measured total efficiencies are from 40% to 80%, the isolation is better than 12 dB, the calculated envelope correlation coefficient (ECC) is less than 0.12, and the calculated channel capacity (CC) ranges from 35 to 38 bps/Hz. The presented antenna array is a good alternative to 5G mobile handsets with wideband operation, a metal frame, and minimized spacing.

## 1. Introduction

Nowadays, various 5G technologies, devices, and applications are being generated as a result of the development of 5G communications. In the context of 5G operational bands, the Third-Generation Partnership Project (3GPP) has defined N77 (3.3–4.2 GHz), N78 (3.3–3.8 GHz), and N79 (4.4–5 GHz) as sub-6 GHz 5G New Radio (NR) frequencies [1,2,3]. In recent years, several dual-band or multi-band antennas capable of covering a fragment of the 5G sub-6 GHz spectrum have been developed [4,5,6,7,8]. The antenna presented in [4] achieves dual-band characteristics by carving an L-shaped slot on the Z-shaped radiation strip and combining it with a parasitic rectangular microstrip. In [5], a folded monopole and an open loop structure are adopted at the top and bottom of the frame to realize the characteristics of dual frequency bands. An E-shaped monopole antenna is presented in [6], where the long side and short side of the E-shaped strip generate the lower and higher frequency bands, respectively. In [7], the current at the higher and lower frequencies is mainly distributed on the left and right sides of an L-shaped patch on a metal rim. A dual-band antenna composed of an outer half-ring strip and two L-shaped strips were superimposed in series, which obtained the lower and higher frequency bands, respectively. In [8], the design consists of four kinds of double-fed loop resonators distributed in four corners of the handset, and strong magnetic fields are distributed on either side of the circular parasitic plane.

Wideband or ultra-wideband handset antennas can efficiently cover the entire 5G sub-6 GHz spectrum, enabling multifunctionality and facilitating large-scale data transmission [9,10,11,12,13,14,15,16,17,18]. In [11], an L-shaped direct-fed patch inside the frame and two L-shaped radiation patches superimposed outside the frame are employed to cover 3.3–5.95 GHz. In [12], a three-dimensional L-shaped feed line and two-dimensional L-shaped metal slot are combined to cover the 5G sub-6 GHz band and WLAN (Wireless Local Area Network) 5 GHz band. In [13], a Y-shaped direct feed strip on the top of the substrate and a shared circular patch on the bottom of the substrate are incorporated to achieve a 121% fractional bandwidth of 2.6–10.2 GHz. Two coupled-loop structures are printed on both sides of the frame, which makes the bandwidth wide enough to cover all of the N77, N78, and N79 frequency bands [14]. In [15], a tuning stub and a meandered line on the feed strip form a -6 dB bandwidth of 3.3–7.1 GHz. In [16], a C-shaped slot on the ground plane, an I-shaped slot on the frame, and a tuning stub are combined to realize an ultra-wideband operation. Although the structure of the antenna branches and parasitic components is complex, it achieved broadband coverage and high efficiency [18]. In [19], the feeding strip connected to the tuning branch effectively excites the double coupled-fed loop modes and a slot mode to operate in the 3.3–6 GHz range.

MIMO antenna technology has been widely used in the design of 5G terminal antennas since it can increase spectral efficiency without excess power consumption and spectrum. However, the restricted space of general-purpose smartphones complicates the design of MIMO antennas. Various decoupling techniques have been presented to achieve high isolation, such as neutralization line structures [20], a T-shaped decoupling stub [21], a rectangular stub with a defected ground plane [21,22], and two H-shaped slots on the ground plane with a fork-shaped microstrip line [23]. In [24,25], four antenna elements are placed at the four corners of the metal frame to realize spatial orthogonal decoupling. In [26], the high isolation of the presented MIMO antenna array is achieved by the combination of quasi-orthogonal polarization, balanced mode excitation, and pattern diversity. A parasitic decoupling structure, aligned with the mesh grid, is specifically designed to improve the isolation between the elements of the MIMO antenna without compromising transparency [27]. This innovative design ensures that the decoupling structure effectively mitigates interference and enhances isolation between the antenna elements while maintaining the desired level of transparency for the antenna configuration.

In this paper, a miniature MIMO antenna array for 5G broadband metal-frame mobile phone applications is proposed. An SRR structure on the frame and a Chinese character Wang-shaped radiator are utilized to achieve a −10 dB bandwidth of 1.7 GHz (3.3–5 GHz), which can cover 5G N77 (3.3–4.2 GHz), N78 (3.3–3.8 GHz), and N79 (4.4–5 GHz). The dimension of the individual antenna element is only 6.8 × 7 × 1 mm^3^ (47.6 mm^3^). The isolation value is larger than 12 dB. Therefore, the innovation points of the proposed handset antenna are wideband operation, high efficiency, and high communication capacity with minimized spacing.

## 2. Structure of the Proposed Handset

**Figure 1:**
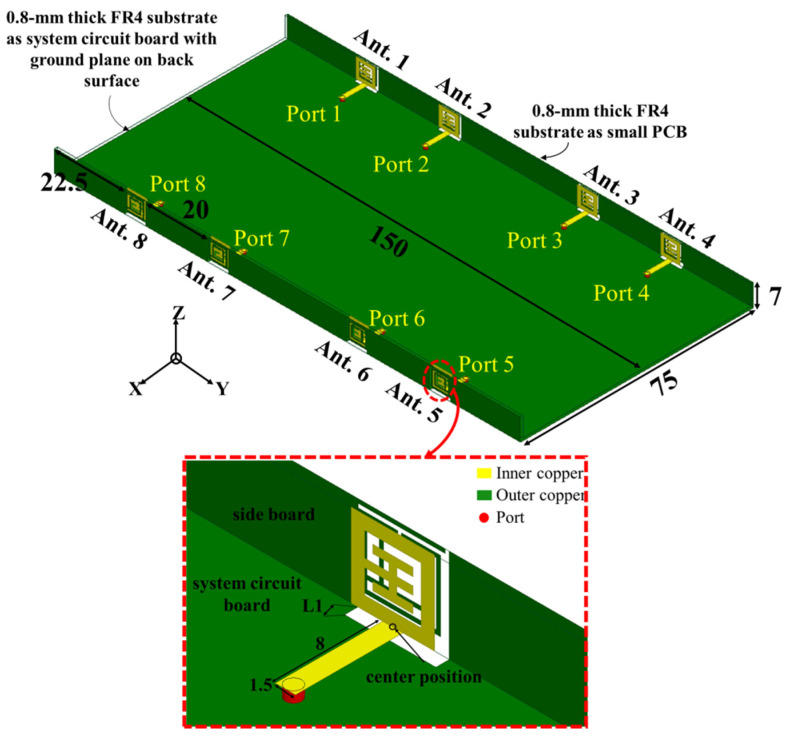

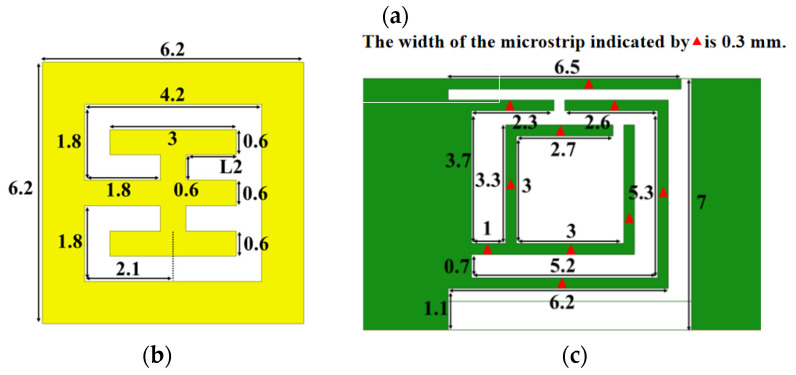
Geometry and dimensions of the proposed wideband eight-element antenna array. (Unit: mm). (**a**) Perspective view. (**b**) Inner copper. (**c**) Outer copper.

The particular geometry and dimensions of the proposed miniature eight-port antenna array are displayed in Figure 1. In Figure 1a, two small PCBs (printed circuit boards) and a system circuit board form a big U-shaped tridimensional substrate that imitates a handset edge frame with a size of 150 × 75 × 7 mm^3^. The thickness of the FR4 (flame retardant 4) substrate is 0.8 mm, the dielectric constant is 4.4, and the loss tan is 0.02. The outside of the two small PCBs and the back side of the system circuit board are grounded with copper. Each small PCB has four antenna elements. The distances between Ant.1 and the frame edge corner, Ant. 1 and Ant. 2, and Ant. 2 and Ant. 3 are 22.5 mm, 20 mm, and 37.8 mm, respectively. Antenna locations are placed according to the worst isolation situation of the antenna array. The Wang-shaped radiator is on the inside of the small PCB and three split-ring resonators. A square frame with a width of 1 mm and external dimensions of 6.2 mm × 6.2 mm encloses the Wang-shaped radiator. The square frame is connected to the Wang-shaped radiator through a short wire at the center of the Wang-shaped feeder unit, facilitating a connection between the inner and outer frames.

**Figure 2:**
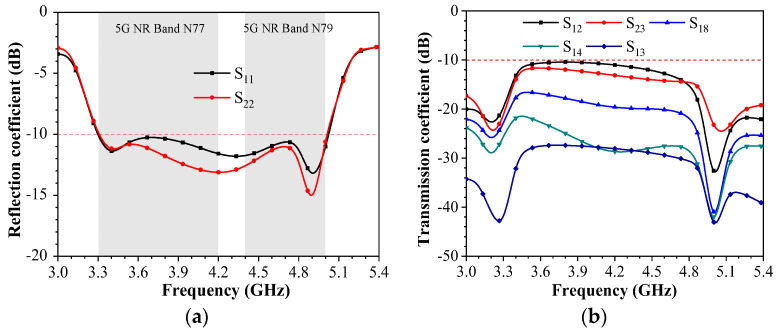
The simulated coefficients of the proposed wideband eight-element antenna array. (**a**) Reflection coefficients. (**b**) Transmission coefficients.

There are three SRRs on the outside of the small PCB. The SSRs are made up of 0.3 mm strips to ensure consistent structure. These strips, as they are bent into various shapes and their sizes change, collaborate with the internal Wang-shaped radiator to achieve functionality. The shapes of the three SRRs are not identical, and their positions correspond to those of the internal Wang-shaped radiating element. The opening positions of each SRR correspond to the internal Wang-shaped radiating element. The width of the feeding line is 1.5 mm, which can achieve the best impedance matching at 3.3–5 GHz. The system circuit board has a cylindrical hole with a radius of 0.6 mm for connecting the SMA (sub-miniature A) probe head from bottom to top. The geometry and detailed dimensions of the proposed wideband antenna element are listed in Figure 1b (inner copper) and Figure 1c (outer copper). The antenna element has a Wang-shaped feed element and an SRR structure on the metal rim, which resonates at 3.5 GHz, 4.3 GHz, and 4.9 GHz, separately. The Wang-shaped feed element is placed at the internal surface of a small PCB. The S-parameter array of the proposed miniature eight-port antenna array is first displayed in Figure 2. The range of reflection coefficients less than −10 dB ranges from 3.3 GHz to 5.02 GHz, while the transmission coefficients are all less than −10 dB within this range. It should be noted that although the MIMO antenna array proposed in this paper has eight antenna elements, each antenna element has identical geometry. According to different antenna element locations, it can be divided into two types, namely Ant. 1 and Ant. 2. Ant. 1, Ant. 4, Ant. 5, and Ant. 8 are all located on the outside of the antenna bezel on each side and, thus, present the same results. Comparatively, Ant. 2, Ant. 3, Ant. 6, and Ant. 7 are located on the inner side of the antenna bezel, which means that these four antennas present the same result. Ant. 1, Ant. 2, Ant. 3, and Ant. 4 are symmetrical about the system. Furthermore, Ant. 5, Ant. 6, Ant. 7, and Ant. 8 are symmetrical with Ant 1, Ant. 2, Ant. 3, and Ant. 4 about the overall system. In addition, the most sensitive isolation can be represented by the couplings between Ant. 1 and Ant. 2, Ant. 2 and Ant. 3, Ant. 1 and Ant. 3, Ant. 1 and Ant. 4, and Ant. 1 and Ant. 8.

## 3. Simulated Result and Analysis

In this section, the evolutionary design, the current distribution, the effects of single-handed holding, and the parameter analysis are deduced to explore the approach employed in designing the featured MIMO antenna using the Ansoft HFSS 15 simulator.

### 3.1. Evolution Design

**Figure 3:**
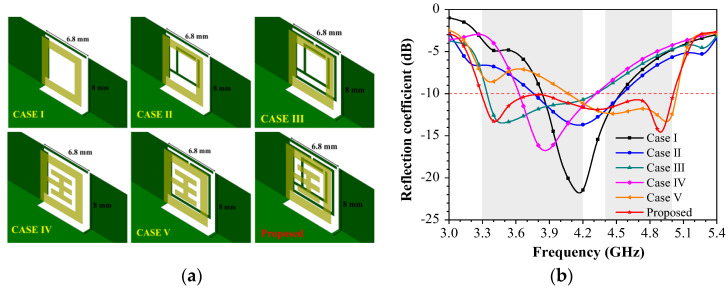
The evolution of the proposed miniature eight-port antenna array. (**a**) Design evolution. (**b**) Reflection coefficients.

Six different cases of antenna elements based on rectangular feed elements (Case I, II, III) and Wang-shaped feed elements (Case IV, V) are discussed, as displayed in Figure 3a. With the rectangular feed element, Case I has a ring resonator on the metal rim, Case II has two ring resonators on the metal rim, and Case III has three ring resonators on the metal rim. The adoption of the SRR structure and Wang-shaped feed element made the width of the antenna element only 6.8 mm. Moreover, it can be found that a wide bandwidth can be obtained by a Wang-shaped feed element and SRR structure on the frame. As displayed in Figure 3b, the −10 dB bandwidth of the proposed miniature eight-port antenna array can cover 3.3–5 GHz.

### 3.2. Parameters Analysis

**Figure 4:**
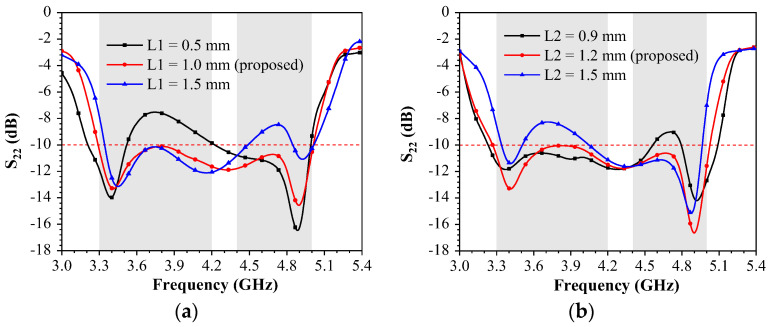
Simulated parameter results of the proposed miniature eight-port antenna array. (**a**) L1. (**b**) L2.

This section mainly investigates the optimization of the parameters of the proposed miniature eight-element MIMO antenna. The effects of the clearing ground distance (L1) on the mainboard and the length of the Wang-shaped feed element (L2) on the sideboard are used to optimize reflection coefficients. Since the proposed miniature eight-port MIMO antenna is symmetrical in structure, antenna elements with the same situation are ignored. In Figure 4a, the reflection coefficient varies as a function of the clear ground distance, L1. To cover both the lower and higher frequency bands, the optimal value of L1 is 1 mm. In Figure 4b, the reflection coefficient varies as a function of the Wang-shaped microstrip line width, L2. When the value of L2 is 1.2 mm, both the lower and higher frequency bands meet the requirement.

### 3.3. Current Distribution

**Figure 5:**
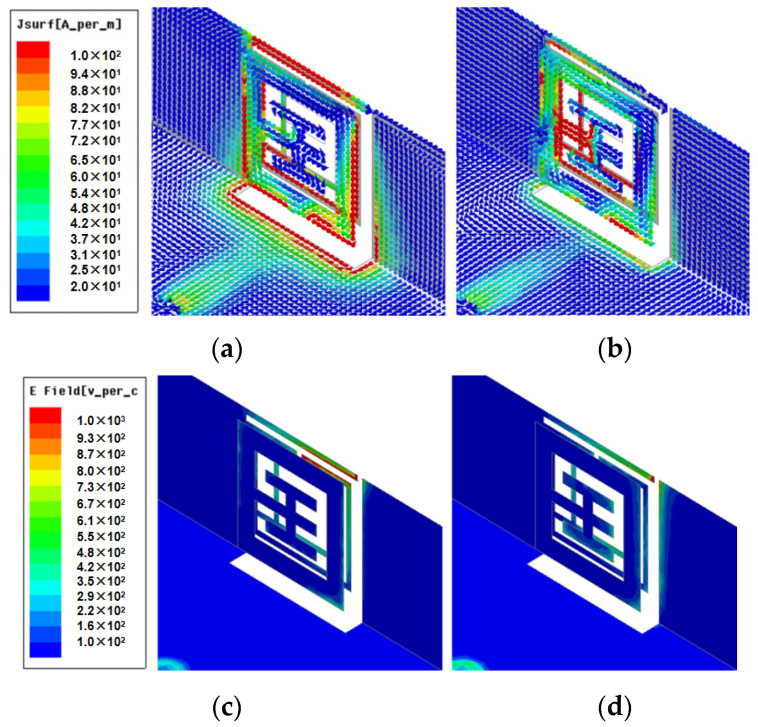
Simulated current and electric field distribution of the proposed miniature eight-port antenna array. (**a**) Current distribution at 3.5 GHz. (**b**) Current distribution at 4.9 GHz. (**c**) Electric field at 3.5 GHz. (**d**) Electric field at 4.9 GHz.

In Figure 5a, the current distribution on the inner surface at 3.5 GHz is primarily focused on the lower left corner of the Wang-shaped feed microstrip line, while the current distribution on the outer surface is concentrated in three open-ring structures with a 0.3 mm width beside the lower left corner. In Figure 5b, the current distribution on the inner surface at 4.9 GHz is primarily centralized in the lower right corner of the Wang-shaped feed microstrip line, while the current distribution on the outer surface is primarily concentrated in the lower right corner of the open-loop structure. In Figure 5c,d, the electric field distribution on the outer surface at 3.5 GHz is primarily focused in the upper right corner of the metal rim, while the electric field distribution on the outer surface at 4.9 GHz is primarily concentrated in the right corner of the metal rim. As a result, three resonant positions constitute a wide frequency band, and the −10dB impedance bandwidth can fully cover 3.3–5 GHz. Therefore, a small variation in the size of the structure can make a big difference in the reflection coefficients. Three open-loop structures can greatly reduce the size of the antenna and achieve wide bandwidth and better antenna performance at the same time, which is very meaningful for the prospect of 5G sub-6 GHz miniature mobile handsets.

### 3.4. Analysis of the Simulated Application Scenario

**Figure 6:**
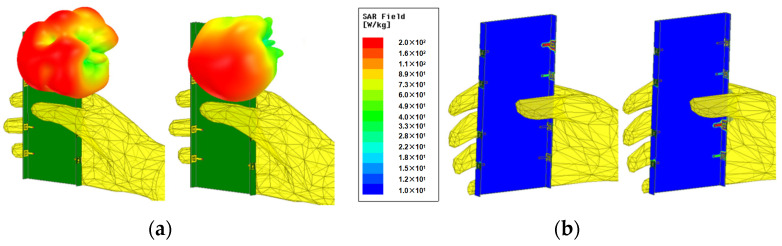
Simulated application scenario of single-handed holding mode. (**a**) Three-dimensional radiation patterns. (**b**) SAR field distributions.

**Figure 7:**
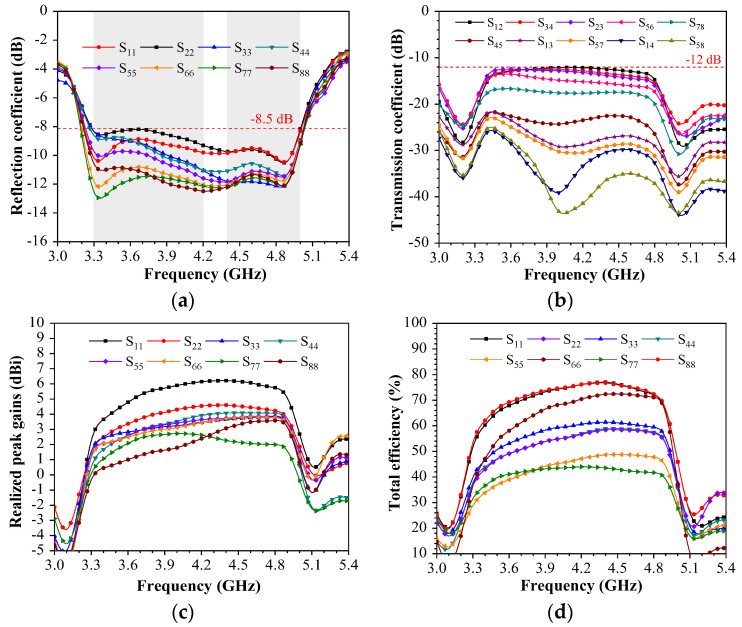
Simulated coefficients of single-handed holding mode. (**a**) Reflection coefficients. (**b**) Transmission coefficients. (**c**) Realized peak gains. (**d**) Total efficiencies.

In Figure 6, one representative application scenario, namely single-handed holding mode (SHM), is presented, and the impact of a hand phantom is studied. The corresponding simulated metrics of the antenna, including S-parameters, the transmission coefficient, the total efficiency, the SAR (specific absorption rate) field, and the 3D radiation pattern, are also shown. For SHM mode, Ant. 3, Ant. 4, Ant. 5, Ant. 6, and Ant. 7 are directly contacted with the hand issue. The 3D radiation pattern of the system and SAR field distribution in SHM mode are shown in Figure 6a,b, respectively. SAR is the power of the electromagnetic waves absorbed or consumed per unit time per unit mass of human tissue. Since smartphone antennas cannot avoid interference from hands, the effects of electromagnetic absorption of the human body can be studied through SAR. The greater the SAR value, the greater the thermal effect of electromagnetic waves on human tissue. The SAR values measured in Figure 6b comply with the Chinese standard SARYD/T 1644.2–2011 [28] body test. Because of this arrangement, the reflection coefficients of these elements deteriorated significantly in both bands, as plotted in Figure 7a. Notably, minimal variation in the transmission coefficient in SHM mode is depicted in Figure 7b. The realized peak gains of the proposed miniature eight-port antenna array, except for Ant. 1, all dropped by 1 dB to 3 dB in the desired bands, as shown in Figure 7c. Since the hand is a lossy medium, the realized peak gains of Ant. 8 also reduced by about 0.8 dB. The total efficiencies of the proposed miniature eight-port antenna array, except for Ant. 1 and Ant. 8, all dropped to between 20% and 40% in the wideband, as shown in Figure 7d. The efficiency of Ant. 5 also reduced to about 45% since the hand is a lossy medium and parts of the radiating power of the antenna element are assimilated by hand.

## 4. Experiment Consequences and Discussion

**Figure 8:**
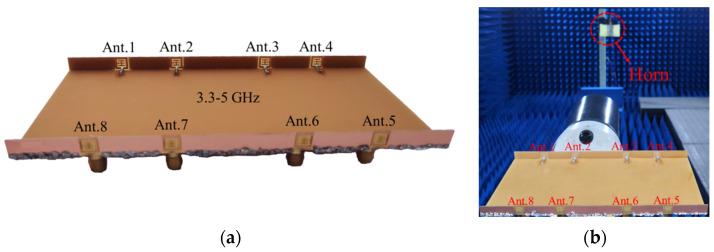
Photograph of a measurement experiment. (**a**) Fabricated antenna prototype. (**b**) Microwave anechoic chamber.

**Figure 9:**
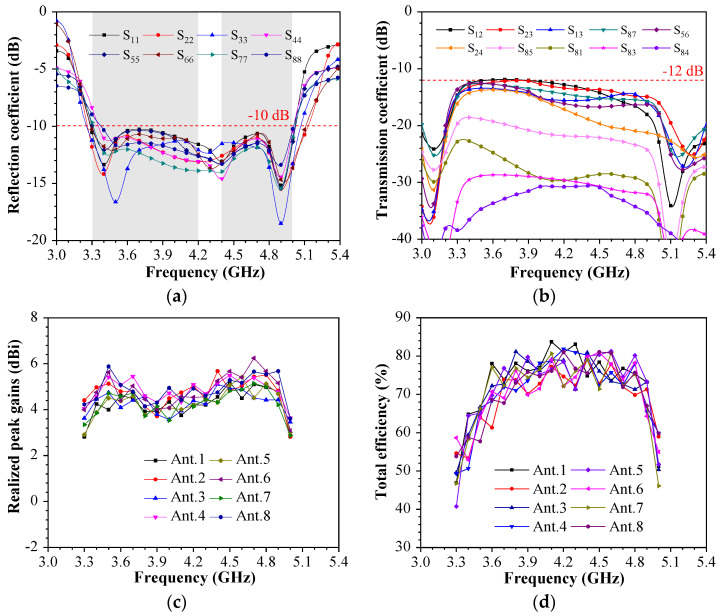
Measured coefficients of the proposed miniature eight-port antenna array. (**a**) Reflection coefficients. (**b**) Transmission coefficients. (**c**) Realized peak gains. (**d**) Total efficiencies.

Figure 8a shows the photograph of a prototype. In Figure 8b, 2D radiation patterns are measured by a microwave anechoic chamber, and the S-parameters are measured through a Keysight vector network analyzer (VNA) with serial number N5224A. The measured S-parameter results are displayed in Figure 9. Figure 9a demonstrates that the proposed miniature eight-port antenna array can operate in a wideband of 3.3–5 GHz with an Snn smaller than −8.5 dB. Generally, the reflection coefficients S11 of mobile handset antennas are expected to be below −6 dB within the specified frequency bands. Figure 9b illustrates the isolation between different antenna elements. The 2 dB improvement in measured isolation values compared to simulation results can be attributed to the reduction in antenna emission, as indicated by Snn < −8.5 dB, potential gaps during the welding of the three substrates, and the presence of excess tin. Additionally, when measuring mobile phone antennas on a vector network analyzer (VNA), susceptibility to interference from the surrounding environment, including instruments, hands, metal desks, etc., should be considered. Figure 9c reveals the realized peak gains, and Figure 9d displays total efficiencies, both of which are crucial measurements conducted in a microwave anechoic chamber.

The calibration of standard horn gain in a microwave anechoic chamber was carried out to obtain anechoic attenuation, including left-hand circularly polarized (LHCP), right-hand circularly polarized (RHCP), co-polarization, cross-polarization, electric field intensity, etc. The measured realized peak gains were from 2.4 dB to 6 dB, and measured total efficiencies were from 40% to 80%.

**Figure 10:**
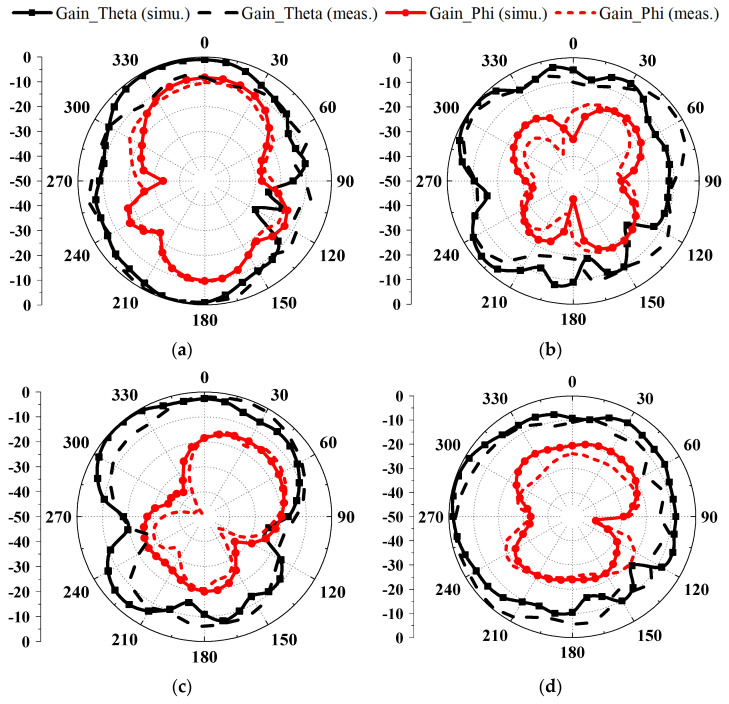

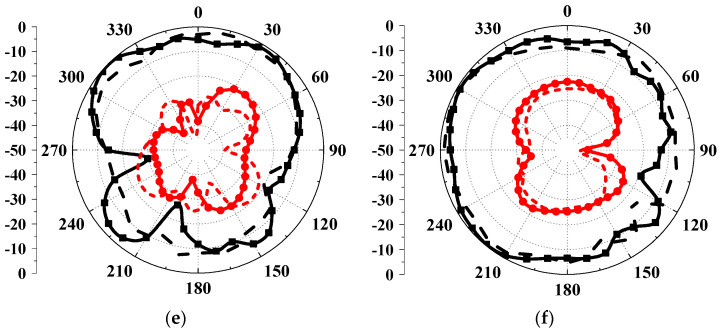
Measured and simulated normalized two-dimensional radiation patterns of the proposed miniature eight-port antenna array: (**a**) 3.5 GHz, E-plane; (**b**) 3.5 GHz, H-plane; (**c**) 4.3 GHz, E-plane; (**d**) 4.3 GHz, H-plane; (**e**) 4.9 GHz, E-plane; (**f**) 4.9 GHz, H-plane.

The measured and simulated normalized two-dimensional radiation patterns of the proposed miniature eight-port antenna array are displayed in Figure 10. The black curves and Ant. 1 are applied to represent the miniature eight-port antenna array with an identical structure. Due to the different locations of Ant. 1 and Ant. 2 on the small PCB, the two-dimensional radiation patterns are slightly different. Due to the regularization from −50 dB to 0 dB, the curve originally presented with a single point of prominence becomes more circular and smoother. In Figure 10, the radiation patterns of simulation and measurement at the same plane and frequency are close to each other. The cross-polarization is smaller than the co-polarization. Some of the cross-polarization displays 8-shaped curves. The measured co-polarization and cross-polarization curves are in good agreement with the simulation.

**Figure 11:**
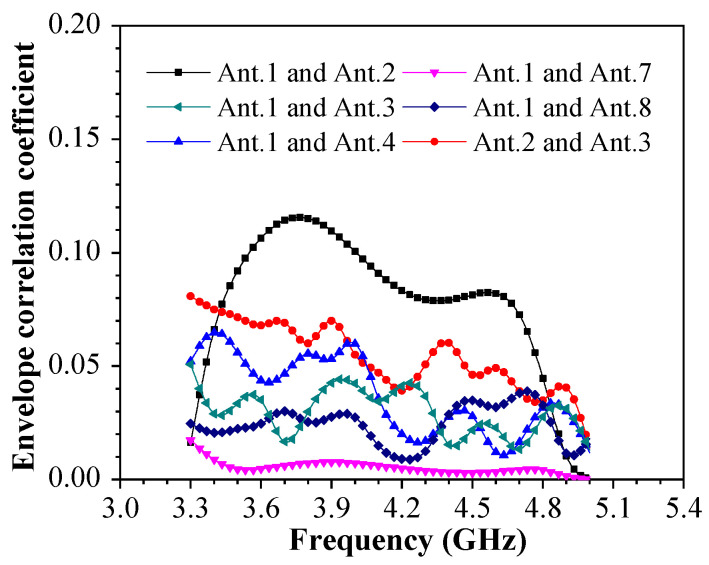
Computational ECC of the proposed miniature eight-port antenna array.

The ECC calculated by the anechoic chamber is relatively more accurate than the ECC calculated directly by the S-parameters. Therefore, the radiation pattern of eight antennas pointed in different directions, combined with high port isolation, should contribute significantly to achieving a smaller ECC value. The MIMO performance of the antenna system will deteriorate with the increase in ECC. The computational ECC of the proposed miniature eight-port antenna array is shown in Figure 11.

(1) $$\rho_{\mathrm{ij}}=\frac{\iint_{4\pi }A_{\mathrm{ij}}(\theta ,\phi )\sin(\theta )d\theta d\phi }{\sqrt{\iint_{4\pi }A_{\mathrm{ii}}(\theta ,\phi )\sin(\theta )d\theta d\phi \iint_{4\pi }A_{\mathrm{jj}}(\theta ,\phi )\sin(\theta )d\theta d\phi }}$$

The acceptable upper limit value of ECC in the general standard is 0.5. The ECC between Ant. i and Ant. j can be calculated by Equation (1), which uses far-field pattern data. The above method is calculated separately in MATLAB or EXCEL, which is also the measurement calculation in practice.

**Figure 12:**
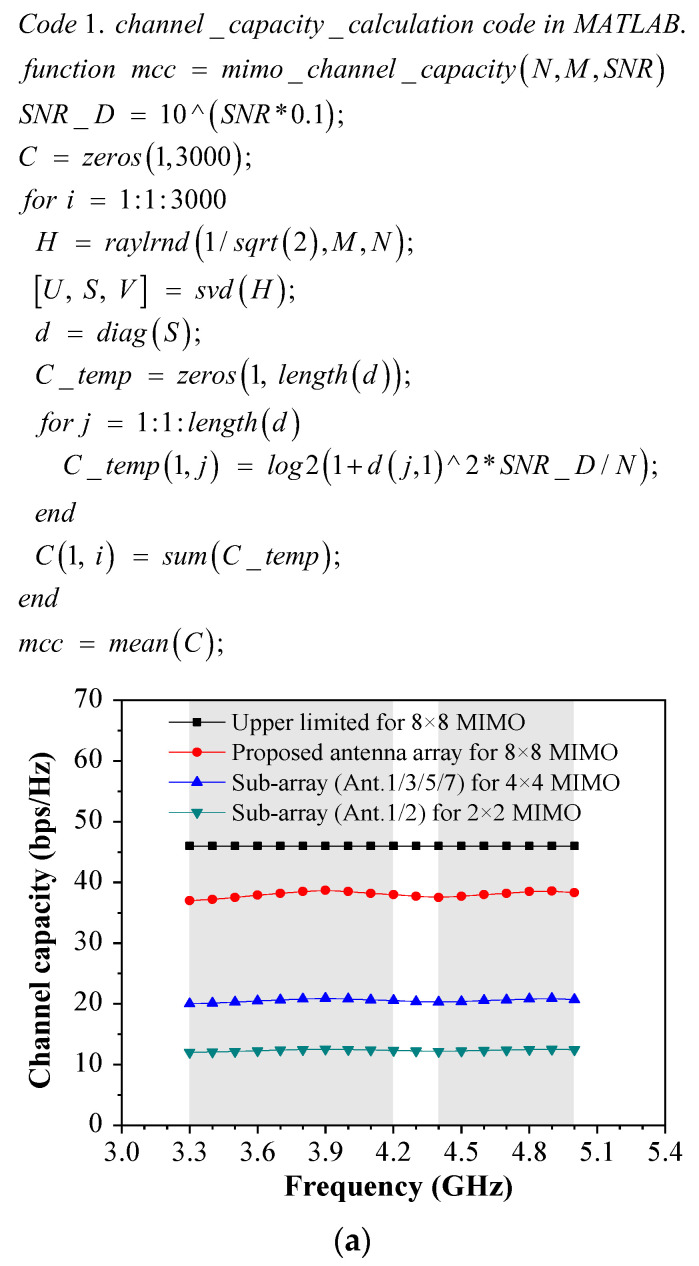

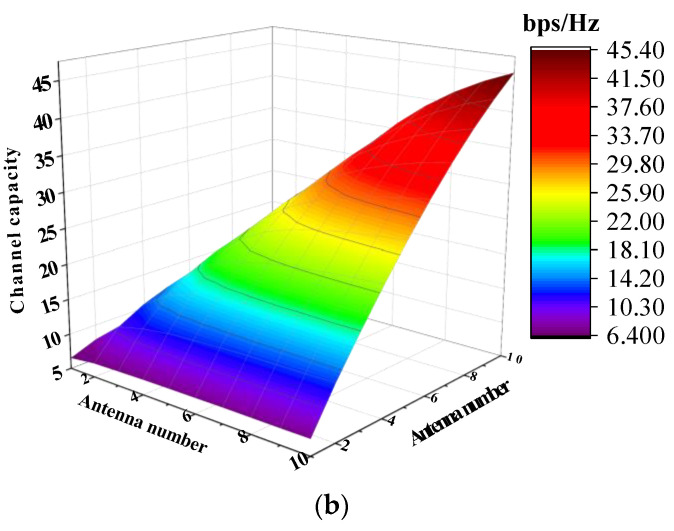
Computational channel capacity as a function of the number of receiving antennas and transmitting antennas. (**a**) Two-dimensional rainbow mesh. (**b**) Three-dimensional rainbow mesh.

In addition to ECC, channel capacity (CC) is also a critical parameter to assess the comprehensive MIMO performance of the proposed miniature eight-port antenna array. The CC is the total channel transmission rate of the MIMO antenna system, and the CC increases with the improvement of antenna element numbers and SNR. The CC can be computed by Equation (2):

(2) $$CC=E\left\{\log_{2}\left[\det\left(I_{N}+\frac{SNR}{N}\right)HH^{T}\right]\right\}$$

(3) $$H=\begin{array}{cccc} h_{11} & h_{12} & \cdots & h_{1Nt}\\ h_{21} & h_{22} & \cdots & h_{2Nt}\\ \cdots & \cdots & \cdots & \cdots \\ h_{Nr1} & h_{Nr2} & \cdots & h_{NrNt} \end{array}$$

where *SNR* is the signal–noise ratio; *E* is the matrix expectation; *N* is the number of antenna elements; *H* is an Nr × Nt identity matrix channel imitating practical space; and HT is the Hermitian transpose of the matrix. The element h_NrNt_ of the H-matrix denotes the transmission channel gain between the transmitting antenna, Nt, and the receiving antenna, Nr. This data-adopted channel is a random array number with a value of 3000. Under the circumstance of best *SNR*, the *CC* of the 4 × 4 MIMO system is 25 bps/Hz, and the 8 × 8 MIMO system is 46 bps/Hz. Code 1 is the channel capacity calculation in MATLAB. The singular value decomposition (SVD) function in code 1 is an orthogonal matrix decomposition method. In the return results, U and V represent two mutually orthogonal matrices, and S represents a diagonal matrix. When the SNR is 22 dB, in Figure 12a, the calculated CC is approximately 35–38 bps/Hz in the desired bands. Figure 12b shows a 3D rainbow mesh of the calculated channel capacity as a function of the number of receiving and transmitting antennas. It can be observed that the channel capacity increases as the number of receiving or transmitting antennas increases. When the SNR is 22 dB, the 4 × 4 antenna number is 22 bps/Hz, the 8 × 8 antenna number is 37.6 bps/Hz, and the 10 × 10 antenna number is 45.4 bps/Hz.

Table 1 compares the MIMO performance of the presented miniature eight-port antenna array and other published papers. Compared with [15], the proposed MIMO antenna array shows smaller element volumes with similar operating frequency bands. The return loss parameter of the present antenna is −10 dB at 3.3–5 GHz, whereas the antenna in [16] exhibits only −6 dB at 3.3–7.1 GHz. This distinction underscores the superiority of the present antenna in terms of this key performance metric. Compared with [17], the proposed antenna has a smaller element volume of 47.6 mm^3^. Compared with other references in Table 1, the smaller element volume is better suited to progressively miniaturize handset devices. The contribution of this work is that it shows wideband operation, small volume, high efficiency, low ECC, and a high CC handset antenna array.

## 5. Conclusions

In this article, a miniature MIMO antenna array for 5G wideband metal-rim mobile phone applications is presented. The individual antenna element consists of a Wang-shaped radiator and three split-ring resonators (SRR) on the metal frame. The dimensions of an individual antenna element are 6.8 × 7 × 1 mm^3^ (47.6 mm^3^). The measured total efficiency ranges from 40% to 80%, the isolation is better than 12 dB, the calculated ECC is less than 0.12, and the calculated CC ranges from 35 to 38 bps/Hz. The innovation points of the proposed handset antenna are wideband operation (which is suitable for 5G sub-6 GHz applications), high efficiency, and high communication capacity under minimized spacing. Other decoupling methods, such as the use of slots and parasitic elements, can be explored to further optimize isolation in future iterations of the antenna design. Therefore, the proposed MIMO antenna array is a good alternative for 5G sub-6 GHz miniature mobile handset applications.

## Figures and Tables

**Table 1:** Performance contrast between the proposed miniature eight-port antenna array and other works.

Reference	−10/−6 dB	ECC	Efficiency (%)	Element Volume (mm^3^)	CC (bps/Hz)
	Bandwidth (GHz)				
[11]	3.3–5.95	<0.11	47–78	81.6	35.6–41.3
	(−10 dB)				
[13]	3.3–6	<0.11	50–82	126	43.93
	(−10 dB)				
[14]	2.6–10.2	<0.007	60–80	1850	40
	(−10 dB)				
[15]	3.3–5	<0.1	40–85	120	31.6–39.2
	(−10 dB)				
[16]	3.3–7.1	<0.09	47–70	44.6	36.5–39.8
	(−6 dB)				
[17]	3.3–6	<0.12	40–71	270	40
	(−6 dB)				
[18]	3.3–6	<0.1	40–70	77.8	39
	(−6 dB)				
This work	3.3–5	<0.12	40–82	47.6	35–38
	(−10 dB)				

## Data Availability

Data are contained within the article.
